# Daily affective experiences are associated with daily, but not trait-level rumination

**DOI:** 10.1192/j.eurpsy.2022.273

**Published:** 2022-09-01

**Authors:** L. Kovacs, N. Kocsel, Z. Toth, T. Smahajcsik-Szabo, S. Karsai, G. Kökönyei

**Affiliations:** 1 Eotvos Lorand University, Institute Of Psychology, Budapest, Hungary; 2 ELTE Eötvös Loránd University, Institute Of Psychology, Budapest, Hungary; 3 Doctoral School of Psychology, ELTE Eotvos Lorand University, Elte, Budapest, Hungary

**Keywords:** rumination on positive affect, daily diary study, daily positive affect, state - and trait-level rumination

## Abstract

**Introduction:**

Rumination is a transdiagnostic risk factor to psychopathology that has mostly been studied in relation to depression (Nolen-Hoeksema, 1991). However, rumination may also occur in response to positive events and emotions (Feldman et al., 2008), and may be a protective factor as it is associated with higher positive affect (Harding et al., 2017).

**Objectives:**

We aimed to examine ruminative response to positive affect (RPA) in daily life and explore its relationship with daily positive and negative affect. We hypothesized that daily positive and negative affect would be associated with daily RPA even after controlling for trait-level RPA and depressive rumination.

**Methods:**

We carried out a daily diary study with university students (n=178). After filling out the baseline survey assessing trait-level rumination, participants had to answer short surveys online about their daily affect and daily rumination every evening for 10 consecutive days. We analyzed our data with multilevel regression in R.

**Results:**

In line with our expectations, daily RPA was significantly associated with daily positive (β=0.16) and negative affect (β=-0.07), while trait-level rumination scores were not significantly associated with daily positive and negative affect. The within-person relationship was stronger between RPA and positive affect (β=0.17) than the between-person relationship (β=0.09). Daily and trait-level rumination were weakly correlated (r=0.218-0.284).

**Conclusions:**

Under ecologically valid conditions, we found that daily rumination was more important in daily affective experiences than trait-level rumination. Understanding whether one’s current affect is more strongly associated with trait-level, state-level or even contextual factors may yield better intervention strategies for affective disorders.

**Disclosure:**

No significant relationships.

